# A mathematical model of lithosphere–atmosphere coupling for seismic events

**DOI:** 10.1038/s41598-021-88125-7

**Published:** 2021-04-21

**Authors:** Vincenzo Carbone, Mirko Piersanti, Massimo Materassi, Roberto Battiston, Fabio Lepreti, Pietro Ubertini

**Affiliations:** 1 Physics Department, Universitá della Calabria, Ponte Pietro Bucci, Rende, Cosenza, Italy; 2 INAF-Istituto di Astrofisica e Planetologia Spaziali, Rome, Italy; 3 Institute of Complex Systems, ISC-CNR, via Madonna del Piano 10, Sesto Fiorentino, 50019 Florence, Italy; 4 Physics Department, Universitá di Trento, Via Sommarive, Povo, Trento, Italy; 5 INFN-TIFPA, Via Sommarive, Povo, Trento, Italy

**Keywords:** Natural hazards, Seismology, Applied mathematics, Fluid dynamics

## Abstract

Significant evidence of ionosphere disturbance in connection to intense seismic events have been detected since two decades. It is generally believed that the energy transfer can be due to Acoustic Gravity Waves (AGW) excited at ground level by the earthquakes. In spite of the statistical evidence of the detected perturbations, the coupling between lithosphere and atmosphere has not been so far properly explained by an accurate enough model. In this paper, for the first time, we show the result of an analytical-quantitative model that describes how the pressure and density disturbance is generated in the lower atmosphere by the ground motion associated to earthquakes. The direct comparison between observed and modelled vertical profiles of the atmospheric temperature shows the capability of the model to accurately reproduce, with an high statistical significance, the observed temperature fluctuations induced by strong earthquakes.

## Introduction

In the last two decades the observation of ionospheric disturbances that precede and follow earthquakes is one of the most debated topic in the literature. The first detection of changes in ionospheric parameters (e.g., F region critical frequency—foF2) associated to the Alaskan earthquake was reported in^1^. In general, it is well known that the ionosphere is influenced from above by the solar activity, leading to the so-called solar wind-magnetosphere-ionosphere coupling^2–5^. On the other hand, the ionospheric medium can be influenced from below by atmospheric waves generated in the neutral atmosphere^6^. Since the principal origin of energy in atmosphere is connected to the its most dense (i.e. lowest) layers, it is expected that ionospheric plasma perturbations could be caused by both dynamic tropospheric process, such as cyclones, motion of weather fronts, jet streams^7,8^, and strong tectonic/technogenic sources of atmospheric oscillations, such as earthquakes, tsunami, volcano eruptions etc.^9–11^. The hypothesis of causal link between ionospheric perturbation observations associated to seismic activity and neutral atmosphere oscillations, leading to Acoustic Gravity Waves (AGW), was first proposed by^12^. AGW indicates one of the dispersion branches in atmospheric waves characterized by a period of approximately 5 minutes to 10 hours, and wavelength of 10 m to 1000 kilometers. Because of the viscous dissipation of the short wave components, its wavelength increases with altitude, reaching hundreds of meters in the ionospheric D layer (located at an altitude between $\sim$ 60 and $\sim$ 90 km) and $\sim 10$ km in the F2 layer (located at an altitude of $\sim$ 400 km). Such waves are the fastest atmospheric oscillations creating upfront of perturbations able to reach the ionosphere^13^. It is worth highlighting that AGWs are able to provide “fast” dynamic coupling between the lower atmosphere and ionosphere, especially those characterized by wavelengths of $\sim 100-200$ km and phase and group velocities of $\sim 100-200$ m/s^8^. In addition, during the last two decades there were several publications on experimental data on AGW induced by the earthquake activity (e.g.^14^, and reference therein).

From a theoretical point of view, waves in the atmosphere, as in any other elastic medium, are a relaxing process that appears in response to any perturbation of equilibrium state of the medium. Atmospheric waves may be described using all theoretical provisions of acoustics of fluids and gases. As a consequence, the oscillations in the atmosphere are described by equations that strictly depends on the pressure, inertia forces, and atmospheric vortexes (cyclones and anticyclones), as result of pressure and Coriolis forces, neglecting the inertia term^15,16^.

In this paper we describe a quantitative model that reproduces with high statistical accuracy how pressure and/or density disturbances can be generated in the lower atmosphere by the ground motion associated to an earthquake and how these disturbances can propagate up to the high atmosphere as AGW. As a case study we have tested the model using data collected during four earthquakes, making a direct comparison between the observed and modelled vertical profiles of the atmospheric temperature fluctuations induced by the different earthquakes.

## Lithosphere: atmosphere coupling model

A seismic event manifests itself through surface waves detected by seismograms, whose dispersion relation is described by Love-Reynolds^17^. The corresponding ground shaking induced by high-magnitude events can excite perturbations at least in the first layer of the atmosphere, that we will call *H*.

Under the hypothesis that the wavelength involved in the perturbation are much grater than *H*, the dynamics of the upper part of the layer can be roughly described within the shallow water approach^18^. For the sake of simplicity we consider a 1D case, and define $\eta (x,t)$ as the fluctuation amplitude of the top of the layer and by *u*(*x*, *t*) the horizontal velocity. In the shallow water framework the time evolution of these quantities can be described by two nonlinear equations that can be cast in a conservative form

1 $$\begin{aligned} \frac{\partial \eta }{\partial t}= & {} - \frac{\partial }{\partial x} \left[ \left( H + \eta - \beta \right) u \right] \end{aligned}$$

2 $$\begin{aligned} \frac{\partial u}{\partial t}= & {} - \frac{\partial }{\partial x} \left( \frac{u^2}{2} + g \eta \right) \end{aligned}$$

where $\beta$ is the “batimetry” of the ground (i.e. the impulsive perturbation of the ground), namely the finite shaking of the ground due to the earthquake. The function $\beta (x,t)$ could be extracted by usual seismograms. However, for the sake of simplicity and without invoking a specific earthquake, we can assume that the impulsive event can be described by a functional shape $\beta (x,t) = \beta _0 f(x,t) w(t)$, where *f*(*x*, *t*) represents the contribution of the seismic surface waves, whose envelope is described by *ω*(*t*), related to the finite duration of the earthquake. Then $f(x,t) \sim \exp [{i(k_sx - \omega _s t)}]$, where $\omega _s/k_s = v_s$ is the phase speed of Love or Rayleigh surface waves, while, looking at a typical seismograph, we can assume a specific functional shape for the envelope $w(t) \sim t \exp ({-\alpha t^2})$, where $\alpha ^{-1/2}$ represents the Strong Motion Duration (SMD) of the seismic event, which is also related to the magnitude.

By using the ansatz where both $\phi (x,t) =[\eta (x,t);u(x,t)]$ are proportional to $\phi (x,t) = \sum _{k,\omega } \phi (k,\omega ) \exp [i(kx - \omega t)]$ on the linearized equations (1), and by considering for simplicity that the seismic event generates surface waves by a single component $(\omega _s,k_s)$, after some algebra we get the surface perturbation at the first atmospheric layer (see “Excited atmospheric modes during large earthquakes”), which can be cast in a recursive form

3 $$\begin{aligned} \eta (k,\omega ) = F(k,\omega ) \eta (k-k_s, \omega - \omega _s) \end{aligned}$$

where the function *F* is reported in “Excited atmospheric modes during large earthquakes”. The solution of (3) allows us to obtain both the normalized amplitude of the perturbation and its dispersion relation (see “Excited atmospheric modes during large earthquakes”). Using Eq. (15) in “Excited atmospheric modes during large earthquakes”, we can obtain the wavemodes $(k,\omega )$ which can be excited by the earthquake.

Once the fluctuations have been generated roughly at the layer $H^\star$ (H* being a characteristic altitude, see "Excited atmospheric modes during large earthquakes"), they give rise to pressure fluctuation. In fact, a parcel of atmosphere at $H^\star$, subject to the vertical displacement $\eta$ from their equilibrium position, obtained from (15), acquire a vertical velocity *w* in a way that the Lagrangian pressure fluctuation is given by^19^

4 $$\begin{aligned} {\tilde{p}} = p - \rho g w \end{aligned}$$

where *p* is the Eulerian pressure perturbation at a fixed point in space. By assuming the harmonic ansatz $\exp [i (\mathbf{k} \cdot \mathbf{r} - \omega t)]$ for fluctuations, where $\mathbf{k} = (k_x, k_y)$, the vertical dependence of both ${\tilde{p}}$ and *w* satisfy a set of first-order ordinary differential equations^20^

5 $$\begin{aligned} \frac{d {\tilde{p}}}{dz} + \frac{gk^2}{\omega _d^2} {\tilde{p}}= & {} \rho \left( \omega _d^2 - \frac{g^2 k^2}{\omega _d^2} \right) w \end{aligned}$$

6 $$\begin{aligned} \frac{dw}{dz} - \frac{g k^2}{\omega _d^2}w= & {} \left( \frac{k^2}{\omega _d^2} - \frac{1}{c_0^2} \right) \frac{{\tilde{p}}}{\rho } \end{aligned}$$

where $c_0$ is the sound speed, $\rho$ is the mass density, and the intrinsic (Doppler-shifted) wave frequency $\omega _d$ is defined as $\omega _d = \omega - \mathbf{k} \cdot \mathbf{u}$, being $\mathbf{u} = (u,v)$ the horizontal velocity. These fluctuations can propagate as an acoustic-gravity wave through the layered atmosphere, and the waveform at a given height *z* above $H^\star$ can be investigated by using the Wentzel-Kramers-Brillouin (WKB) approximation^21^ (see “Propagation of fluctuations through atmosphere: a WKB approach”), thus obtaining the pressure fluctuations at a given height in the atmosphere. As can be seen from Eq. (18) in “Excited atmospheric modes during large earthquakes”, the dispersion relation of wave-vectors/frequencies, excited at height, $H^\star$ strictly depends on specific earthquake parameters, namely: the Peak Ground Acceleration (PGA), the length of the fault (L), the Strong Motion Duration (SMD - $\alpha$), the dominant seismogram frequency ($\omega _s$) and the phase speed of the surface waves ($v_s$).

## Discussion

**Figure 1:**
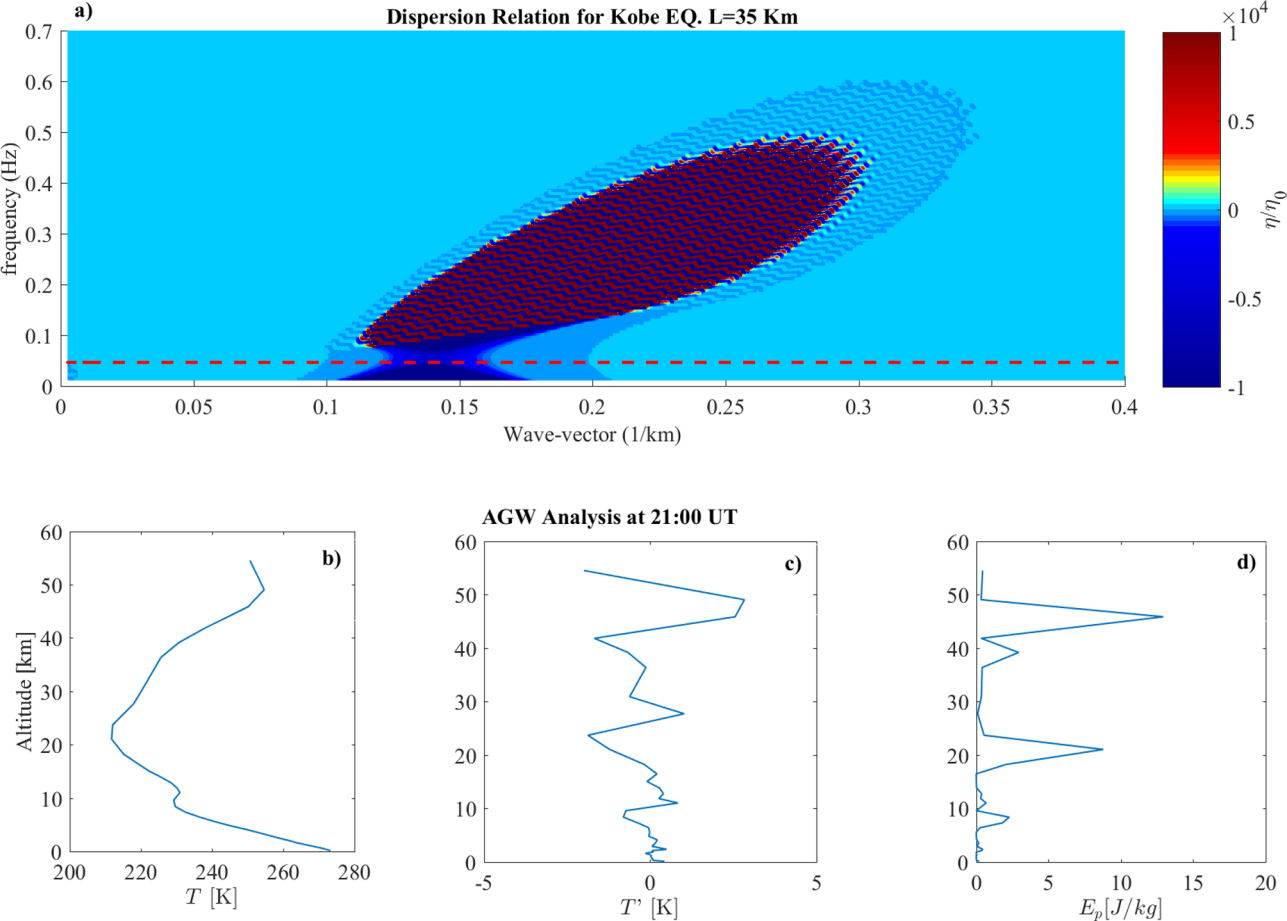
(**a**) Dispersion Relation evaluated for the Kobe Earthquake using the parameters in Table 1. The dashed line represents the parameter $c_0/2h$. (**b**) Vertical Temperature profile over the EQ epicenter. (**c**) Fluctuations of the vertical temperature profile over the EQ epicenter. (**d**) Potential energy density vertical profile over the EQ epicenter.

**Figure 2:**
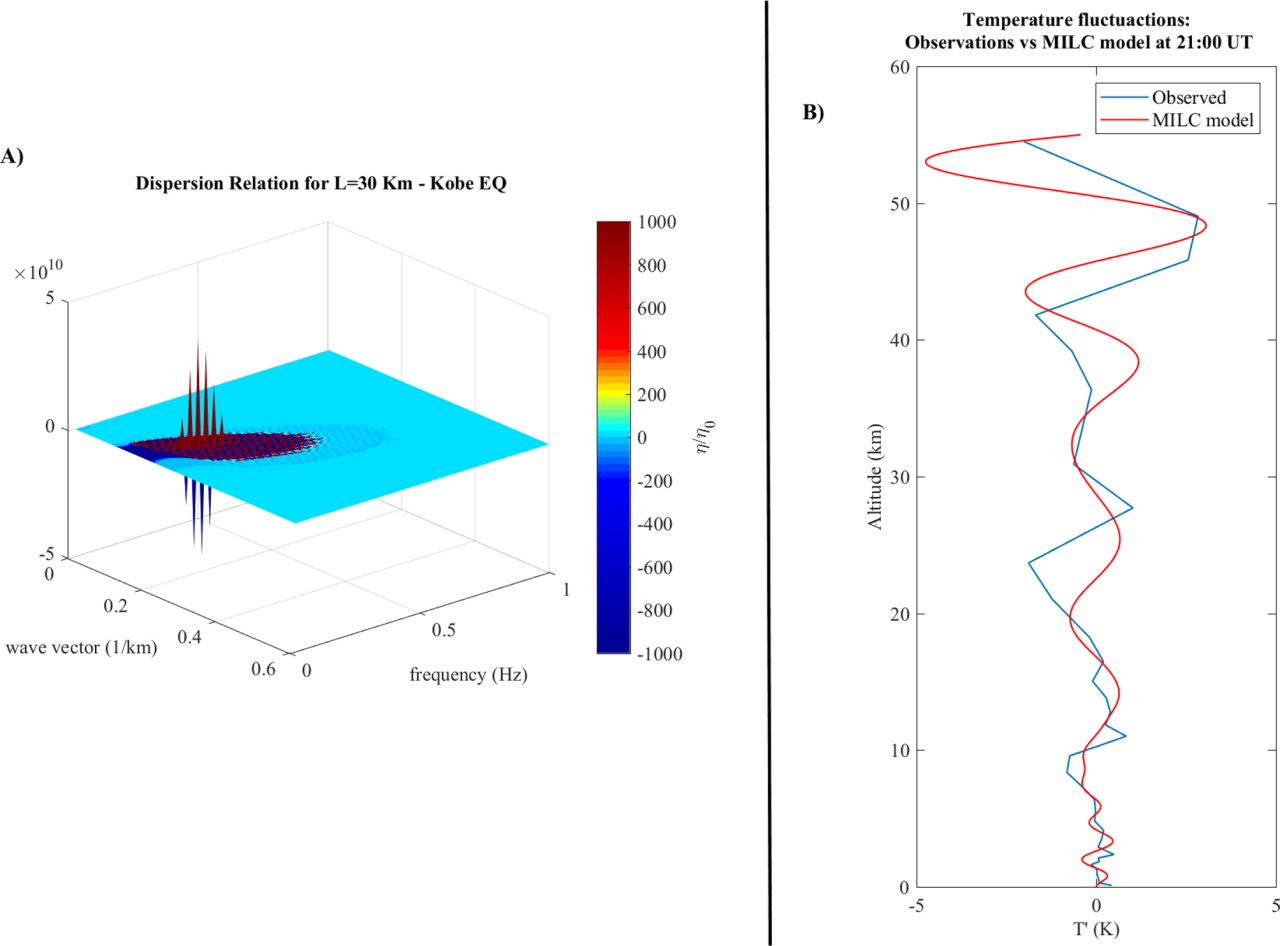
Box (**A**) Tri-dimensional dispersion relation evaluated for the Kobe Earthquake using the parameters in Table 1. The dashed line represents the parameter $c_0/2h$. Box (**B**) Fluctuations of the vertical temperature profile over the EQ epicenter (blue line) superimposed to the modelled temperature previsions at 21:00 UT.

**Figure 3:**
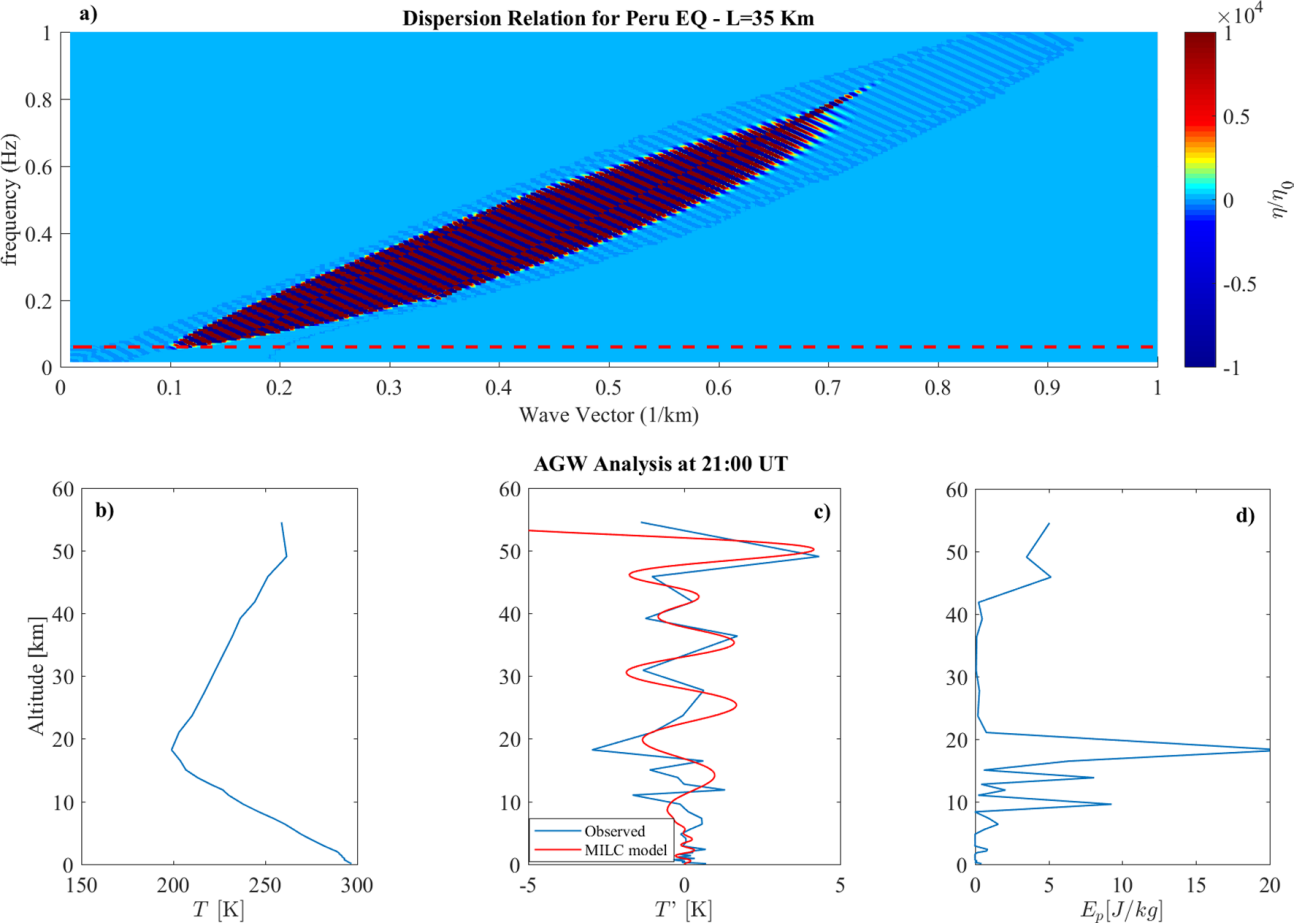
(**a**) Dispersion Relation evaluated for the Peru Earthquake using the parameters in Table 1. The dashed line represents the parameter $c_0/2h$. (**b**) Vertical Temperature profile over the EQ epicenter. (**c**) comparison between fluctuations of the vertical temperature profile observed (blue line) and modelled (red line) over the EQ epicenter. (**d**) Potential energy density vertical profile over the EQ. epicenter.

**Figure 4:**
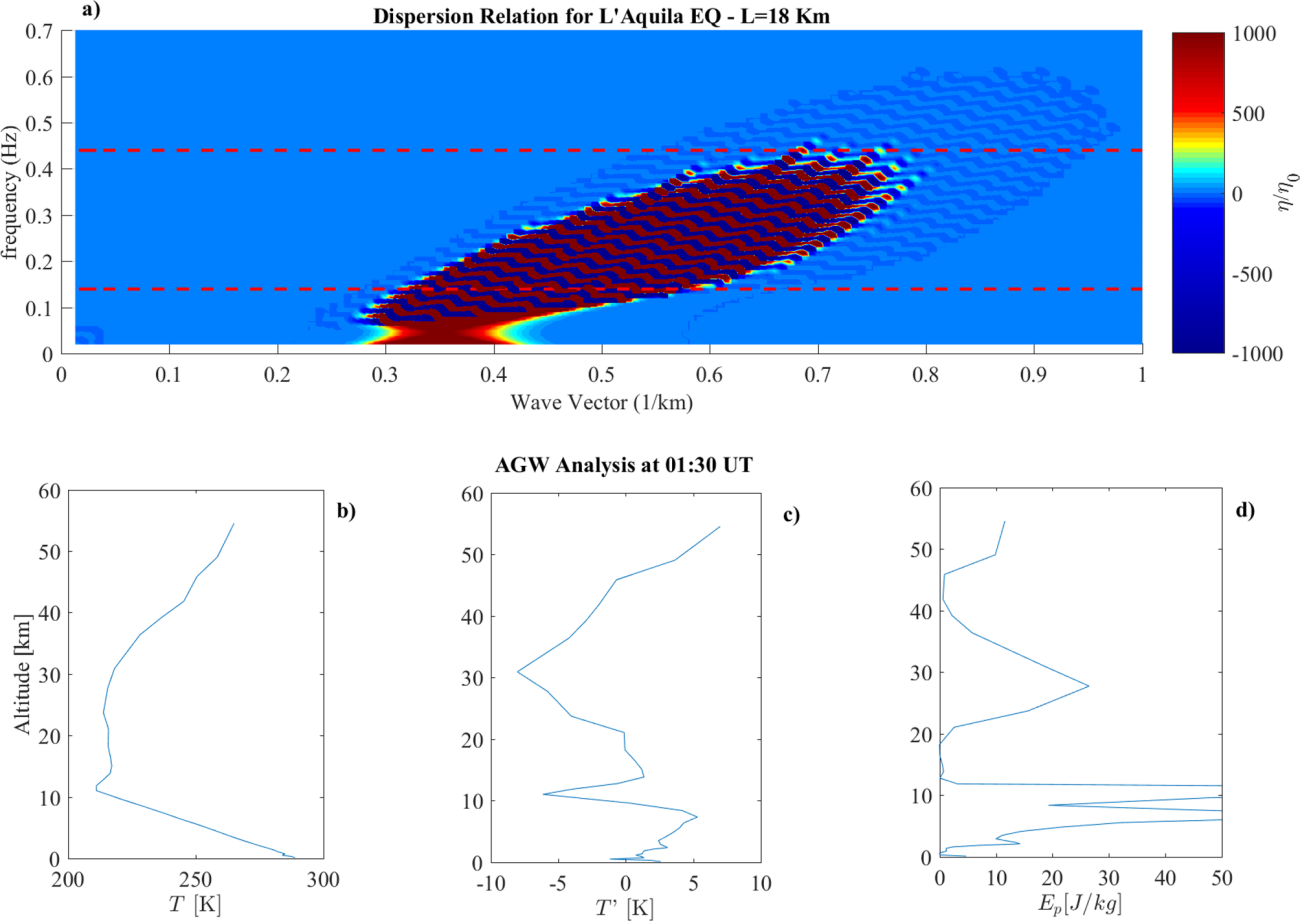
(**a**) Dispersion relation evaluated for the L’Aquila Earthquake using the parameters in Table 1. The dashed line represents the parameter $c_0/2h$. (**b**) Vertical Temperature profile over the EQ epicenter. (**c**) Fluctuations of the vertical temperature profile over the EQ epicenter. (**d**) Potential energy density vertical profile over the EQ.

**Figure 5:**
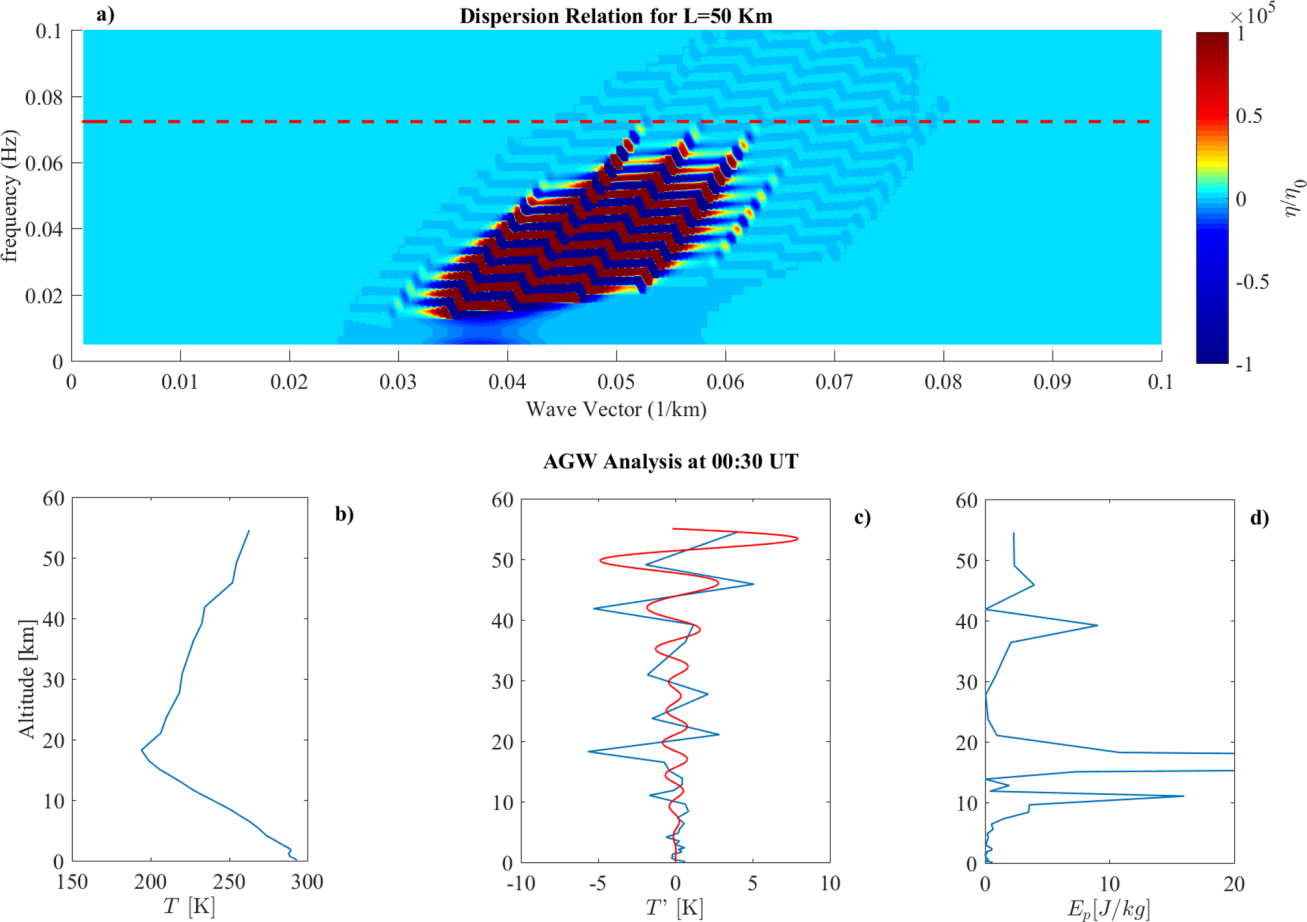
(**a**) Dispersion Relation evaluated for the Fiji Earthquake using the parameters in Table 1. The dashed line represents the parameter $c_0/2h$. (**b**) Vertical Temperature profile over the EQ epicenter. (**c**) Fluctuations of the vertical temperature profile over the EQ epicenter. (**d**) Potential energy density vertical profile over the EQ.

We can investigate the dispersion relation wave-vectors/frequencies excited by a specific earthquake at height $H^\star$. As case studies we have investigated four earthquakes whose characteristic parameters are reported in Table 1. We obtained the earthquake parameters from the USGS dedicated website (www.usgs.gov/natural-hazards/earthquake-hazards/earthquakes). The dispersion relations of fluctuations for each earthquake, in the plane $(k,\omega )$, are reported in panel (a) of Figs. 1, 2, 3, 4 and 5. Experimental observations of AGW eventually detected over the earthquake epicenter are reported in panels (b), (c) and (d) of Figs. 1, 3, 4 and 5.

**Table 1:** Parameters used for the evaluation of the pressure fluctuations dispersion relations associated to 1998 Kobe, 2001 Peru, 2009 L’Aquila and 2018 Fiji earthquakes.

	Date	Magnitude	L (km)	$\omega _s$ (Hz)	PGA (g)	$\alpha$ (s)	$v_s$ (km/s)	$k_s \cdot 10^{-5}$ (1/m)	$H*$ (m)	$\omega _t$ (Hz)
Kobe	16/01/95	6.9	35	0.058	0.8	20	4.8	1.2	338	0.044
Peru	23/06/01	8.2	35	0.036	0.3	40	1.6	2.2	203	0.051
L’Aquila	06/04/09	5.9	18	0.05	0.5	33	1.5	3.3	395	0.43
Fiji	19/08/18	8.2	50	0.01	0.77	35	4.5	0.2	161	0.072

Figure 1 shows the results obtained for 1995 Kobe earthquake. Looking at the dispersion relation it can be seen that fluctuations $\eta (k,\omega )$ have been roughly excited for wave-vectors ranging from $0.1 \le k \le 0.35$ km$^{-1}$, well above $k_s$, and frequencies $0.1 \le \omega \le 0.6$ Hz well above $\omega _s$. Red dashed line in the panel a) represents the threshold ($\omega _t=c_0/h$, h being the temperature scale height) for the pressure fluctuations to propagate or do not propagate throughout the atmosphere up to the ionosphere, as purely vertical AGW, i.e. with ${\mathbf {k}} \cdot {\mathbf {u}} \simeq 0$. In fact, as a consequence of Eq. (24), waves at frequencies $\omega$ excited by the seismic event, which satisfies $\omega ^2>\omega _t^2$, are not evanescent and can propagate to high atmosphere. We evaluated $\omega _t=0.044 Hz$ using the temperature profile retrieved from ERA5, which is the $5^{th}$ generation atmospheric data set produced by the European Centre for Medium-Range Weather Forecasts^22^. As a result of our model, all the excited modes of $\eta$ (panel a) can propagate up to the ionosphere. So we expected to observe AGW injection in conjunction with the seismic event.

In order to check for possible AGW emission over the earthquake epicenter, we applied the method of Piersanti et al. [2020]^11^. The results are presented in panels b), c) and d), displaying vertical atmospheric temperature profile, the atmospheric temperature fluctuations with respect to a 3 km running average and the atmospheric potential energy density, respectively, as obtained from ERA-5 database. A clear AGW propagation, characterized by $\sim 2$ km and $\sim 6$ km wavelength, is detected in conjunction with the Kobe earthquake occurrence^11^.

Box (B) in Fig. 2 shows the direct comparison between the observed (blue line) and the modelled (red line) temperature fluctuations vertical profile. The latter one can be estimated from the pressure fluctuations model by using the equation of gas in atmosphere ${\tilde{p}}(z) = \rho (z) R^* T'(z)$, and a model profile for the atmospheric density $\rho (z) = \rho _0 \exp (-\gamma z)$, where $R^*=R/M$, *R* is the gas constant, $M=28.97$ g is the atmospheric mean molecular mass, $\rho _0=1 km/m^3$ and $\gamma =0.06$ was atmospheric density mass decay index^23^. The evaluation of the model temperature was done by solving the Eq. (26) in “Propagation of fluctuations through atmosphere: a WKB approach” for wave-vectors and frequencies obtained by the dispersion relation (Fig. 2 Box A). In particular we used the three pairs corresponding to the maximum values of $\eta /\eta _0$ (see Table 2) and T’(0)=0 as a boundary conditions.

**Table 2:** Parameters ($k - \omega$) used to model the temperature fluctuations over KOBE and PERU earthquake epicenter.

	Pair 1	Pair 2	Pair 3	RMSE (K)	$\rho _{corr}$
**KOBE**					
*k* ($$km^ {-1})$$	0.53	0.21	0.17	0.8	0.86
$\omega$ (*Hz*)	0.026	0.197	0.163		
**PERU**					
*k* ($$km^ {-1})$$	0.8	0.48	0.11	0.5	0.88
$\omega$ (*Hz*)	0.51	0.43	0.15		

The model is able to correctly reproduce the temperature fluctuation observations with a RMSE (root mean square error) of 0.8 K and a correlation coefficient of 0.86. In addition, to determine whether there is a statistically significant difference between the temperature fluctuations measured and the modelled one, we have performed a $\chi ^2$ test (see Table 3 in “[Sec Sec9]”), obtaining $\chi ^2=47.3$, suggesting that our model is able to reproduce the observations with $>90\%$ probability. It is interesting to highlight that any phase shift due to a ${\mathbf {k}} \cdot {\mathbf {u}}$ transverse wave-vector increases the $\chi ^2$ value. As a consequence, the pure vertical propagation represents the *minimum* $\chi^2$ condition.

The same analysis has been repeated for the 23 June 2001 Peruvian earthquake. Looking at the dispersion relation (Fig. 3a), we can see that fluctuations $\eta (k,\omega )$ have been roughly excited with wavevectors ranging from $0.1 \le k \le 0.72$ km$^{-1}$, well above $k_s$, and frequencies $0.05 \le \omega \le 0.8$ Hz well above $\omega _s$. Also in this case our model expected an AGW injection in conjunction with the earthquake occurrence, since all the excited modes of $\eta$ are greater than $\omega _t=0.051\,Hz$. This prevision is confirmed by the experimental vertical temperature profile and potential energy density (panels b), c) and d). As for the Kobe event, we modelled the vertical behaviour of the temperature fluctuation using the three pairs corresponding to the maximum values of $\eta /\eta _0$ (see Table 2). Even in this case, the model is able to correctly reproduce the temperature fluctuation observations with a RMSE of 0.5 K and a correlation coefficient of 0.88. The $\chi ^2$ test confirms again that the model is able to reproduce the observations with > $90\%$ probability with a $\chi ^2$=48.8. Also in this case, we obtained that the pure vertical propagation represents the *minimum* $\chi^2$ condition.

The third event analyzed is the 6 April 2009 L’Aquila earthquake, where ionospheric disturbances were not observed. The analysis of experimental vertical temperature profile (Fig. 4b–d) confirms the lack of AGW injection over the earthquake epicenter. Figure 4a displays the dispersion relation, where $\eta (k,\omega )$ have been roughly excited for wavevectors ranging from $0.28 \le k \le 0.8$ km$^{-1}$, well above $k_s$, and frequencies $0.08 \le \omega \le 0.44$ Hz well above $\omega _s$. However, in this case, all the excited modes are lower than $\omega _t=0.43 Hz$: in this case possibly generated AGW are evanescent thus preventing a purely vertical propagation into the high atmosphere.

The analysis of the 19 august 2018 Fiji earthquake shows some peculiar features. In fact, the hypocenter of the seismic event is situated at a large depth, about 300 km, and we observe that fluctuations at the first layer in atmosphere (Fig. 5) have been excited for values of wave-vectors and frequencies lower than the previous cases we analyzed. In particular, the modes excited have wave-vectors of the order of few tens of meters, and frequencies of the order of a few tens of mHz, of the order of the surface waveform $k_s$ and $\omega _s$ (Fig. 5a). The evaluation of $\omega _t \simeq 0.072$ Hz, suggests that, in this case, the purely vertical propagation of AGW cannot be excited as a consequence of the earthquake occurrence. However, experimental observations (Fig. 5b–d) show an AGW propagation concurrently with the earthquake occurrence. In this case the discrepancy with our model prediction is simply related to the absence of a purely vertical propagation. In fact, when we allow a phase shift due to a weak transverse wave-vector, that is ${\mathbf {k}} \cdot {\mathbf {u}} \simeq 0.49$ Hz, the excited fluctuation (corresponding to the pair: $k_1$=0.72 $km^{-1}$ and $\omega _1$=0.43 *Hz*) can propagate as a quasi-vertical AGW, and the temperature profile is almost correctly reproduced by our model even in this particular case (Fig. 5c). In this conditions the RMSE is 2.8K and the correlation coefficient $\rho =0.72$. Finally, for the Fiji earthquake the $\chi ^2$ test gives worse result with reference to previous events. In fact, we obtained $\chi ^2$=62.3, corresponding to 65$\%$ probability of the model to reproduce the Fiji temperature fluctuations.

## Conclusion

The possibility to correctly model, for the first time with high statistical significance, the lithosphere–atmosphere coupling, i.e. how the pressure and density disturbance propagates in the upper atmosphere in case of large earthquakes, is a breakthrough in the co- and post-earthquakes analysis. In particular, modelling how strong earthquakes can excite perturbations in the lower atmosphere that propagates vertically as AGW, allows a robust statistical analysis that, in turn, provide a unprecedented tool to trim key parameters to fully reproduce the observed perturbations. In addition, the model allows the a-priori simulation of earthquake effects in the high atmosphere just during and after the event, and opens new scenarios to investigate, through a realistic physical model, co-seismic ionosphere disturbances due to high-magnitude earthquakes. This is a first step towards forecasting earthquake effects in area known to be subjected to large release of energy triggered by tectonic, volcanic etc, events.

More specifically, we have modelled the atmospheric fluctuations excited by a generic seismic event on the top of the first layer of the atmosphere, and estimate its dispersion relation as a function of the characteristic parameters of the earthquake. Then, using the Wentzel-Kramers-Brillouin (WKB) approach^21^, we model the pressure fluctuations of the AGW excited by these near-ground fluctuations. The proposed model is able to provide correct descriptions of AGW emission for three superficial earthquakes. For two of them, where the AGW are expected to propagate and are actually observed^11^, the model provide a direct comparison between computed and observed atmospheric vertical temperature profiles. It shows high reliability, with $90\%$ of probability, to correctly reproduce the temperature fluctuations induced by the earthquake with a low RMSE and correlation coefficient larger than 0.86. The model is also able to correctly reproduce the case when ionosphere disturbances were not observed, i.e. L’Aquila earthquake, showing that AGW should be evanescent.

**Figure 6:**
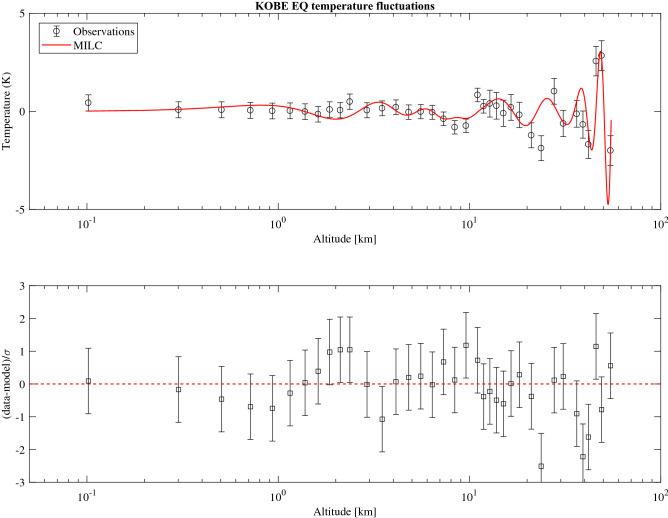
The figure shows, as an example, the comparison between observed (black circle) and modelled (red line) fluctuations of the vertical temperature profile for KOBE EQ (Top panel); In the bottom panel are shown the residuals with respect the model in 1$\sigma$ units. As can be seen the model follows very well the data profile as a function of the altitude with a good statistical approximation.

The choice of an analytical model, despite the possible lower accuracy with respect to a numerical one, allows to control and understand the physical process behind the atmosphere-lithosphere coupling system during active seismic conditions. Finally, as shown in Fig. 6 for the KOBE case, the good statistical agreement between the model and the data will allow to trim the model taking into account different earthquakes features, by the use of a minimum $\chi ^2$ technique, while available a large data set, with small statistical error and low systematic uncertainties.

## Methods

### Excited atmospheric modes during large earthquakes

Let us consider the linearized equations (1)

7 $$\begin{aligned} \frac{\partial \eta }{\partial t} + H \frac{\partial u}{\partial x}= & {} u \frac{\partial \beta }{\partial x} + \beta \frac{\partial u}{\partial x} \end{aligned}$$

8 $$\begin{aligned} \frac{\partial u}{\partial t} + g \frac{\partial \eta }{\partial x}= & {} 0 \end{aligned}$$

where, as well known, it is evident that the batimetry modifies the usual nondispersive oscillations described by the classical relations dispersion $\omega ^2/k^2 = v_0^2 = Hg$. By assuming a flat soil, let us suppose now that a seismic event modify impulsively the “batimetry” $\beta (x,t)$, which represents here the shacking of the ground during the earthquake. Introducing the Fourier series

9 $$\begin{aligned} \phi (x,t) = \sum _{k,\omega } \phi (k,\omega ) \exp [i(kx - \omega t)] \end{aligned}$$

where $\phi$ means each of the variables $\eta$, *u* and $\beta$, from the linearized equations we get a pair of algebraic equations

$$\begin{aligned}&i \sum _{k,\omega } \left[ \omega u(k,\omega ) - k g \eta (k,\omega ) \right] e^{i(kx-\omega t)} = 0 \\&\quad -i\sum _{k,\omega } \left[ \omega \eta (k,\omega ) - k H u(k,\omega ) \right] e^{i(kx-\omega t)} \\&\quad = \sum _{p,w} u(p,w) e^{i(p x-w t)} \sum _{q,r} iq \beta (q, r) e^{i(q x-r t)} \\ & \qquad + \sum _{p,w} ip u(p,w) e^{i(p x-w t)} \sum _{q,r} \beta (q, r) e^{i(q x-r t)} \end{aligned}$$

The Fourier coefficient of $\beta (x,t)$ can be easily calculated

10 $$\begin{aligned} \beta (k,\omega ) = \beta _0 e^{i(kx - \omega t)} \frac{\pi ^{1/2}}{4 \alpha ^{3/2}} \omega e^{-\omega ^2/4 \alpha } \delta _{k, k_s} \delta _{\omega , \omega _s} \end{aligned}$$

so that the sum over the pair (*q*, *r*) can be eliminated by the delta functions, and, after anti-transforming, we obtain the two following equations for the Fourier coefficients

$$\begin{aligned} u(k,\omega )= & {} \frac{gk}{\omega } \eta (k,\omega ) \\ k H u(k,\omega ) - \omega \eta (k,\omega )= & {} \pi ^{1/2} \frac{ \beta _0 k \omega _s}{4 \alpha ^{3/2}} e^{-\omega _s^2/4 \alpha } u(k-k_s,\omega -\omega _s) \end{aligned}$$

By inserting $u(k, \omega )$ into the second equation, we obtain a single equation for the perturbation coefficients which can be cast in the recursive form

11 $$\begin{aligned} \eta (k,\omega ) - F(k,\omega ) \eta (k-k_s, \omega -\omega _s) = 0 \end{aligned}$$

where the multiplicative factor is defined as

12 $$\begin{aligned} F(k,\omega ) = \frac{\pi ^{1/2}}{4} \left( \frac{\beta _0}{v_0} \right) \frac{\omega _s e^{-\omega _s^2/4 \alpha }}{\alpha ^{3/2}} \left( \frac{\omega }{k v_0} \right) \left( 1- \frac{\omega ^2}{k^2 v_0^2} \right) ^{-1} \end{aligned}$$

By interpreting *k* and $\omega$ in terms of the ground values $\kappa = k/k_s$ and $\zeta = \omega /\omega _s$, we can rewrite eq. (11) as

13 $$\begin{aligned} \eta (\kappa ,\zeta ) - \psi (H, \beta ,\alpha ,v_s) \left\{ \frac{\gamma (\zeta /\kappa )}{\left[ 1- \gamma ^2 (\zeta /\kappa )^2\right] } \right\} \eta (\kappa -1, \zeta -1) = 0 \end{aligned}$$

where $\gamma = v_s/v_0$, the function $\psi$ contains only parameters of the earthquake

14 $$\begin{aligned} \psi (H, \beta ,\alpha ,v_s) = \frac{\sqrt{\pi g}}{4} \frac{\beta }{ \sqrt{H}} \left( \frac{\omega _s}{\alpha }\right) e^{-\omega _s^2/4 \alpha } \end{aligned}$$

and $\beta = \beta _0/ g \sqrt{\alpha }$ is a dimensionless parameter.

The factorized form of Eq. (13) is particularly useful, because it can be solved recursively starting from a reference value $\eta _0 = \eta (0,0)$ which represents the unperturbed background at the surface eight *H*. Let us consider a length scale *L* for wavevectors, and a time scale *T* for frequencies, then they can be enumerated by using $k = (2\pi /L) n$ and $\omega = (2\pi /T)m$, where the pair $(n,m) = 0, \pm 1, \pm 2, \dots$ are the integers of the wavevector-frequency plane. The shallow water approach requires $L>> H$, say $kH<< 1$. If the earthquake generates surface waves with a length scale $\lambda$, the above condition becomes $k/k_s<< \lambda /H$. This implies that, for a given lengthscale of surface waves, the perturbation can be generated not so far from the ground and wavevectors can be not so high.

If the earthquake produces surface waves with length scale $\lambda$ and time scale *P*, we have $\kappa _n = (\lambda /L) n$ and $\zeta _m = (P/T)m$. Iterating relation (13), the Fourier coefficients at a given wavelength-frequency $(\kappa _n, \zeta _m)$, are then described by

15 $$\begin{aligned} \eta (\kappa _n,\zeta _m) = \eta _0 \left[ \psi (H,\beta ,\alpha ,v_s)\right] ^{nm} \Gamma (\kappa _n,\zeta _m) \end{aligned}$$

where, by fixing a pair (*i*, *j*) on the plane and defining

$$\begin{aligned} v_{i,j} = \left( \frac{P}{T}\right) \left( \frac{\lambda }{L}\right) \left( \frac{v_s}{v_0}\right) \left( \frac{j}{i}\right) \end{aligned}$$

we have

16 $$\begin{aligned} \Gamma (\kappa _n,\zeta _m) = \prod _{i=1}^n \prod _{j=1}^m \left[ \frac{v_{i,j}}{1- v_{i,j}^2}\right] \end{aligned}$$

### Dispersion relation

First of all note that the parameter $\beta$ can be interpreted as the Peak Ground Acceleration (PGA) of the earthquake which, in general, is estimated from seismograms in terms of *g*. This implies that the function $\psi$ contains the information on the characteristic of earthquake, the only unknown quantity is the height *H* of the layer of atmosphere we are considering. Since the relation (13) is a recursive equation, we must avoid divergences as well as very low values of the perturbation. We can estimate a characteristic height $H^\star$ where the value of the amplitude can induce an efficient perturbation for each mode $(k,\omega )$ from the relation $\psi (H^\star ,\beta ,\alpha ,v_s) \simeq 1$, that is

17 $$\begin{aligned} H^\star \simeq \frac{\pi g}{16} \beta ^2 \left( \frac{\omega _s}{\alpha }\right) ^2 \exp {(-\omega _s^2/2 \alpha )} \end{aligned}$$

We can interpret this as follows. If we assume that the earthquake is able to induce perturbations leading to acoustic-gravity waves in the atmosphere, the typical amplitude of the perturbation can be roughly excited at least at a characteristic height of the order of $H^\star$, according to relation (17). Assuming $H = H^\star$, the function $\Gamma$ defined by (16), represents a kind of dispersion relation, in the plane $(\kappa _n, \zeta _m)$, which gives information on the waveforms excited by the seismic event.

As a rough estimate we can use $P \sim \omega _s^{-1}$, while the time base can be estimate as the earthquake duration, say $T \sim 1/\sqrt{\alpha }$. On the other hand, since $\lambda>> H^\star$, we can assume $\lambda = r H^\star$, where *r* is a free parameter, and *L*, the length base, can be taken to be as an estimate of the seismic faulting. In this way

18 $$\begin{aligned} v_{i,j} = \left( \frac{\sqrt{\alpha } H^\star }{\omega _s}\right) \left( \frac{r}{L}\right) \left( \frac{v_s}{v_0}\right) \left( \frac{j}{i}\right) \end{aligned}$$

and the dispersion relation depends on the free parameters *r* and *L*. In our calculations of the dispersion relation from real earthquakes, we used $r = 10$.

### Propagation of fluctuations through atmosphere: a WKB approach

In the WKB framework, assuming that the scale height of atmosphere *h*, the velocity $\mathbf{u}$ and the sound speed $c_0$ are smooth function of $z/\Lambda$, where $\Lambda$ represents some scale of variation of the variables, as a first-order approximation the Fourier coefficient of pressure fluctuations at a given height *z* is related to that at $H^\star$ through^21^

19 $$\begin{aligned} p(z) = \frac{\omega _d^2 \pm img - g/2h}{\omega _d^2 - g^2 k^2 \omega _d^{-2}} {\tilde{p}}(z) \end{aligned}$$

where

20 $$\begin{aligned} {\tilde{p}}(z)= & {} {\tilde{p}}(H^\star )\sqrt{\frac{m(H^\star )A(z)}{m(z)A(H^\star )}} \exp \left[ \pm i \left( \int _{H^\star }^z m(z^\prime ) dz^\prime + \xi \right) \right] \end{aligned}$$

21 $$\begin{aligned} \xi= & {} \int _{H^\star }^z \frac{dz^\prime }{2m} \left[ \frac{gk^2}{\omega _d^2} \frac{d}{dz^\prime }\ln \left( \frac{h}{\omega _d^4-g^2k^2}\right) + \frac{1}{2h} \frac{d}{dz^\prime } \ln \left( \frac{\omega _d^4 - g^2 k^2}{\omega _d^2} \right) \right] \end{aligned}$$

the vertical wavevector is defined as:

22 $$\begin{aligned} m^2 + \left( \frac{1}{2h} - \frac{gk^2}{\omega _d^2} \right) ^2 = \left( 1-\frac{g^2 k^2}{\omega _d^4}\right) \left( \frac{\omega _d^2}{c_0^2}-k^2\right) \end{aligned}$$

and the coefficient

23 $$\begin{aligned} A = \rho \left( \omega _d^2 -\frac{g^2 k^2}{\omega _d^2}\right) \end{aligned}$$

When the condition of propagation $m^2 > 0$ is satisfied, either the upper and lower sign in (20) propagate as oblique local plane waves. As $m^2 < 0$ the wave is evanescent, while $m^2({\tilde{z}})=0$ defines the so-called turning points ${\tilde{z}}$ where WKB approximation fails. Moreover, WKB approximation diverges at points where either $\omega _d=0$ or $\omega _d^2 = kg$. However, neither of these conditions exist for purely vertical propagation when $k=0$^21^. If we consider quasi-vertically propagating waves, by assuming $k<< 1$, from (22) we obtain

24 $$\begin{aligned} {m}^2 \simeq \left( \frac{\omega ^2}{c_0^2} - \frac{1}{4h^2}\right) - \left( \frac{2 \omega }{c_0^2}\right) \mathbf{u}\cdot \mathbf{k} + 0(k^2) \end{aligned}$$

Purely vertical propagation requires that frequencies $\omega$ excited by the seismic event must satisfies $\omega ^2 > c_0^2/4h^2$, in order to be not evanescent and can propagate to high atmosphere. Under this condition, the pressure fluctuations associated to quasi-vertically propagating waves, according to WKB, are

25 $$\begin{aligned} p(z) \simeq \frac{\omega ^2 \pm i {m}g - g/2h}{\omega ^2} {\tilde{p}}(z) \end{aligned}$$

where

26 $$\begin{aligned} {\tilde{p}}(z) \simeq {\tilde{p}}(H^\star )\sqrt{\frac{{m}(H^\star )\rho (z)}{{m}(z)\rho (H^\star )}} \exp \left[ \pm i \int _{H^\star }^z {m}(z^\prime ) dz^\prime \right] \end{aligned}$$

Equations (25) and (26) represents vertically propagating acoustic-gravity waves, with vertical wavevector (24), generated from the perturbation of frequency $\omega$ excited at height $H^\star$ by ground motion due to the large earthquake.

### $\chi ^2$ test

Here we show the table for the evaluation of the $\chi ^2$ test for the KOBE earthquake event and the comparison between the modelled and the observed atmospheric temperature fluctuations (Fig. 6).

**Table 3:** Temperature fluctuations observed vs modelled for the Kobe earthquake.

Altitude (km)	$T'_{observed} (K)$	$T'_{model} (K)$	$(T'_{observed}-T'_{model})^2 (K^2)$
54.59	$-$1.996	$-$1.919	0.005929
49.04	2.837	2.765	0.005184
45.84	2.557	1.0575	2.24850025
41.82	− 1.683	− 0.0454	2.68173376
39.17	− 0.671	1.072	3.038049
36.36	− 0.1293	0.5258	0.42915601
30.9	− 0.6216	− 0.5639	0.00332929
27.71	1.023	0.5922	0.18558864
23.69	− 1.879	0.3999	5.19338521
21.04	− 1.221	− 0.5355	0.46991025
18.23	− 0.1801	− 0.2988	0.01408969
16.48	0.2026	0.1239	0.00619369
15.04	− 0.09765	0.338	0.189790923
13.83	0.2817	0.5249	0.05914624
12.77	0.3952	0.4273	0.00103041
11.85	0.2606	0.2227	0.00143641
11.02	0.836	0.03857	0.635894605
9.582	− 0.7331	− 0.6579	0.00565504
8.368	− 0.8103	− 0.3217	0.23872996
7.317	− 0.382	− 0.368	0.000196
6.389	− 0.04751	− 0.009013	0.001482019
5.56	− 0.0165	− 0.09194	0.005691194
4.809	− 0.04025	− 0.08866	0.002343528
4.124	0.2105	0.05253	0.024954521
3.494	0.1537	0.4659	0.09746884
2.910	0.05543	0.02691	0.00081339
2.367	0.4936	− 0.2144	0.501264
2.109	0.05807	− 0.3863	0.197464697
1.859	0.09172	− 0.3475	0.192914208
1.616	− 0.1471	− 0.2124	0.00426409
1.381	− 0.01452	− 0.02012	0.00003136
1.153	0.03035	0.1232	0.008621123
0.9313	0.02111	0.3072	0.081847488
0.7156	0.05147	0.3021	0.062815397
0.5056	0.07786	0.2190	0.0199205
0.3011	0.07984	0.1021	0.000495508
0.1017	0.4315	0.1389	0.08561476
